# Supplementary material for: Phenotypic and genomic changes in enteric Klebsiella populations during long-term ICU patient hospitalization: the role of RamR regulation
Source: mSphere. 2024 Nov 29;9(12):e00704-24. doi: 10.1128/msphere.00704-24 (PMC11656808; doi:10.1128/msphere.00704-24)
Supplement: Figure S1 — Patient hospitalization timelines. [file msphere.00704-24-s0001.pdf]

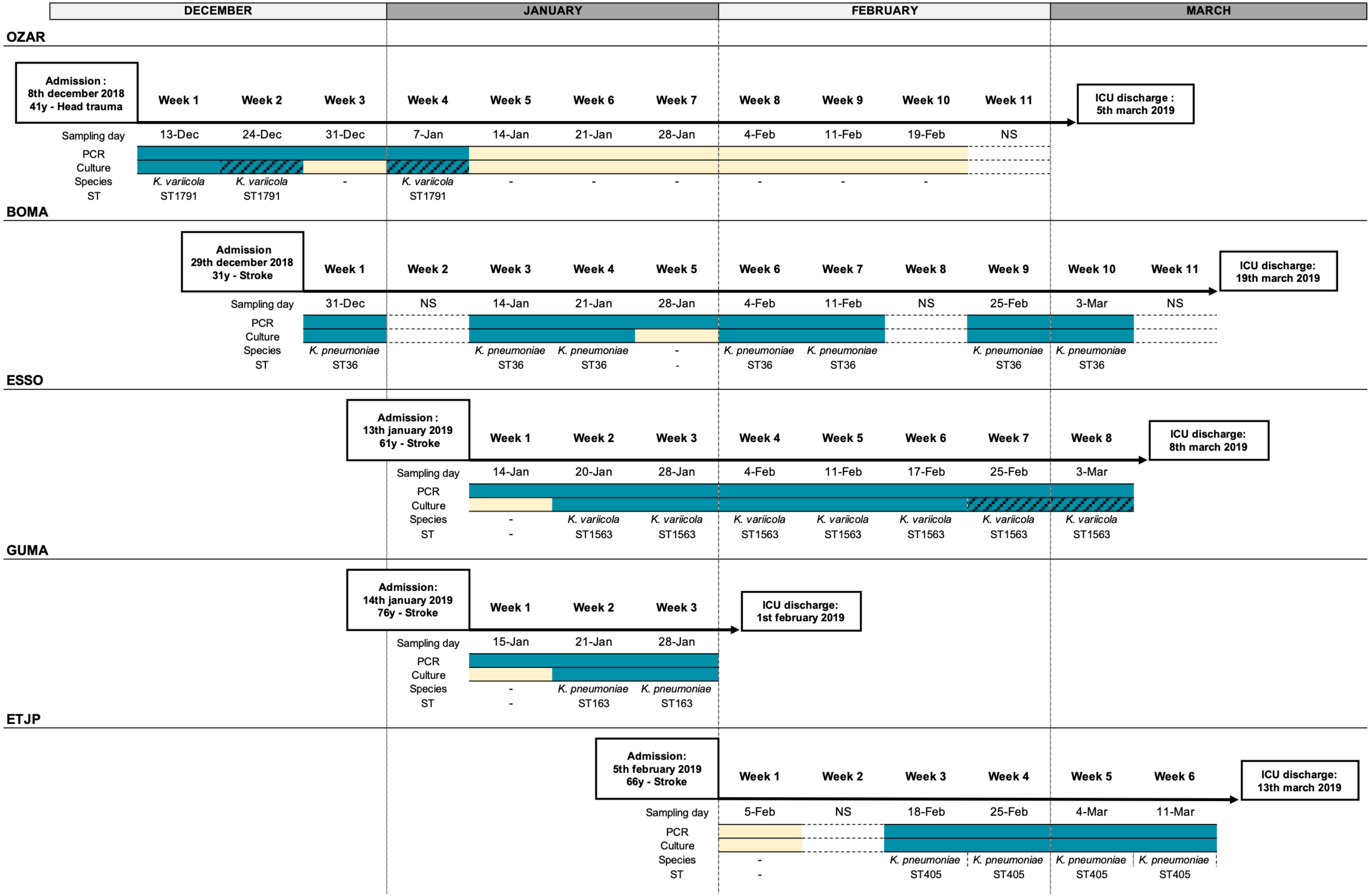

Figure S1: Patient hospitalization timelines.
NS: No Sample; blue case: positive, yellow case: negative; dashed case: resistance acquisition
