# Supplementary material for: Phenotypic and genomic changes in enteric Klebsiella populations during long-term ICU patient hospitalization: the role of RamR regulation
Source: mSphere. 2024 Nov 29;9(12):e00704-24. doi: 10.1128/msphere.00704-24 (PMC11656808; doi:10.1128/msphere.00704-24)
Supplement: Figure S2 — Growth curves for ESSO strains. [file msphere.00704-24-s0002.pdf]

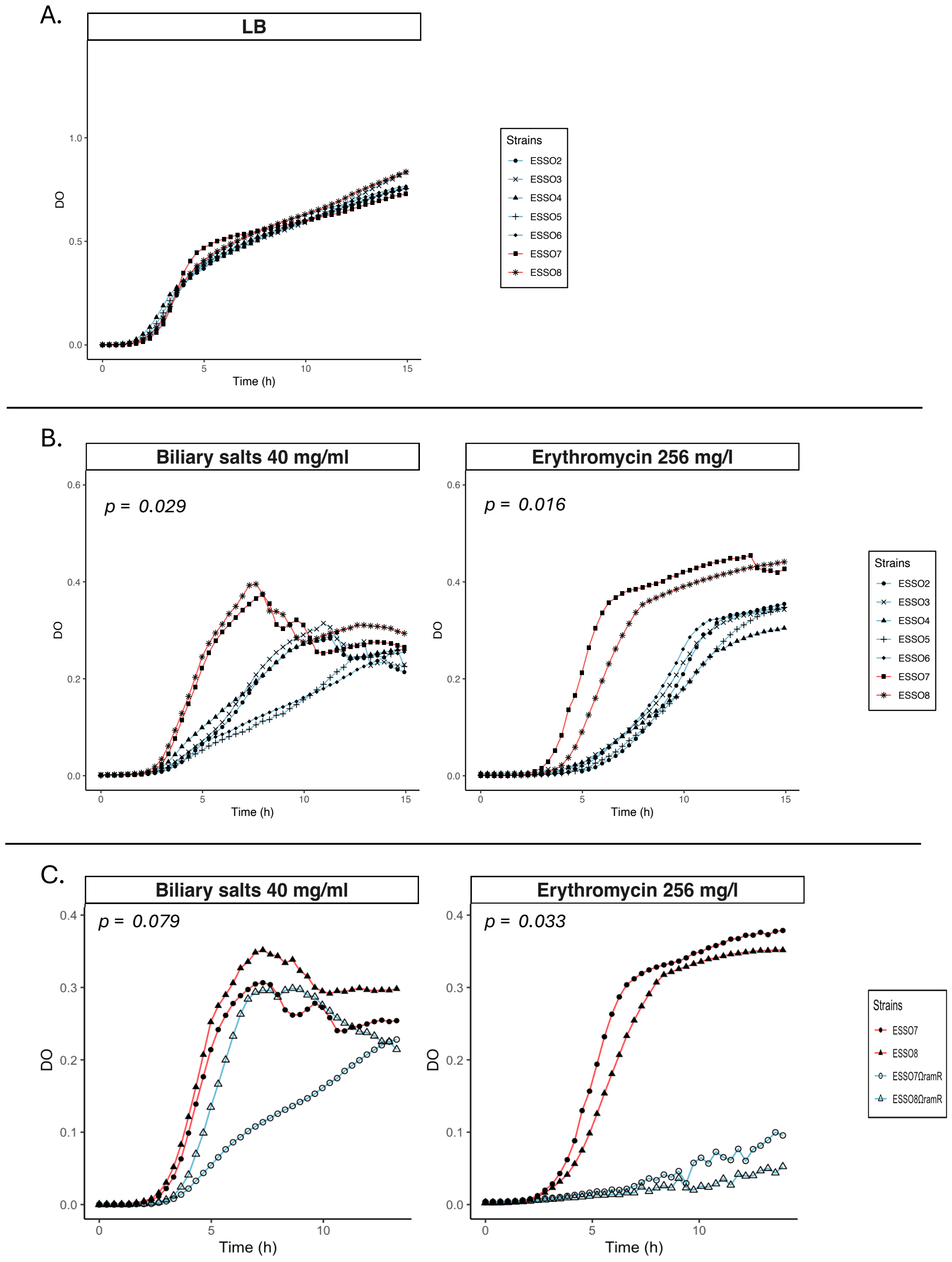

Figure S2: Growth curves for ESSO strains. *p.val*ue calculated with student test indicates significance of generation time between red and blue curves.

A. Growth curves for all ESSO strains - Blue lines for not mutated *ramR K. variicola* strains and red line for *ramR* mutated strains in LB

B. Growth curves for all ESSO strains - Blue lines for not mutated *ramR K. variicola* strains and red line for *ramR* mutated strains in presence high concentrations of biliary salts or erythromycin

C. Growth curves for complemented ESSO strains - Blue lines for complemented *ramR K. variicola* strains and red line for *ramR* mutated strains in presence high concentrations of biliary salts or erythromycin
