# Supplementary material for: Phenotypic and genomic changes in enteric Klebsiella populations during long-term ICU patient hospitalization: the role of RamR regulation
Source: mSphere. 2024 Nov 29;9(12):e00704-24. doi: 10.1128/msphere.00704-24 (PMC11656808; doi:10.1128/msphere.00704-24)
Supplement: Tables S1, S2, and S5 — Antibiotics, antiseptic ranges, and primers. [file msphere.00704-24-s0003.docx]

**Supplementary material and methods Appendix**

**Table S1: name, concentration and suppliers of the antibiotics used for the antimicrobial susceptibility profile determination, using disk diffusion method**

| **Antibiotic** | **Disk charge (µg)** | **Supplier** |
| --- | --- | --- |
| ampicillin | 10 µg | Bio-Rad, France |
| amoxicillin-clavulanate | 30 µg | Bio-Rad, France |
| ticarcillin | 75 µg | Bio-Rad, France |
| ticarcillin-clavulanate | 85 µg | Bio-Rad, France |
| piperacillin-tazobactam | 36 µg | Bio-Rad, France |
| cefalexin | 30 µg | Bio-Rad, France |
| cefamandole | 30 µg | Bio-Rad, France |
| cefoxitin | 30 µg | Bio-Rad, France |
| ceftriaxone | 30 µg | Bio-Rad, France |
| ceftazidime | 10 µg | Bio-Rad, France |
| cefepime | 30 µg | Bio-Rad, France |
| ceftolozane-tazobactam | 40 µg | Bio-Rad, France |
| ertapenem | 10 µg | Bio-Rad, France |
| imipenem | 10 µg | Bio-Rad, France |
| meropenem | 10 µg | Bio-Rad, France |
| temocillin | 30 µg | Bio-Rad, France |
| mecillinam | 10 µg | Bio-Rad, France |
| aztreonam | 30 µg | Bio-Rad, France |
| amikacin | 30 µg | Bio-Rad, France |
| gentamycin | 10 µg | Bio-Rad, France |
| tobramycin | 10 µg | Bio-Rad, France |
| netilmicin | 10 µg | Bio-Rad, France |
| streptomycin | 10 µg | Bio-Rad, France |
| spectinomycin | 100 µg | Bio-Rad, France |
| nalidixic acid | 30 µg | Bio-Rad, France |
| ciprofloxacin | 5 µg | Bio-Rad, France |
| norfloxacin | 10 µg | Bio-Rad, France |
| levofloxacin | 5 µg | Bio-Rad, France |
| azithromycin | 15 µg | Bio-Rad, France |
| tigecycline | 15 µg | Bio-Rad, France |
| tetracycline | 30 µg | Bio-Rad, France |
| fosfomycin | 200 µg | Bio-Rad, France |
| polymyxin B | 300 UI | Bio-Rad, France |
| chloramphenicol | 30 µg | Bio-Rad, France |
| rifampicin | 5 µg | Bio-Rad, France |

**Table S2: Ranges used for antiseptic susceptibility testing**

| **Antiseptic** | **Range** | **Supplier** |
| --- | --- | --- |
| alcoholic chlorhexidine 2% | 256 to 1 mg/L | Gilbert laboratories, France |
| povidone-iodine 10% (Betadine®) | 50 000 to 195 mg/L | Meda Pharma, France |
| didecyldimethylammonium chloride (DDAC) | 256 to 1 mg/L | Anios, France |

**Table S5: Primers used for qRT-PCR experimentations**

| **Primer** | **Sequence (5’ -> 3’)** |
| --- | --- |
| Ram_pBAD202_F | CACCCGGTTCATATCCTGACCAGA |
| Ram_pBAD202_R | CTCGGTAAACGGGTAGGTCA |
| GyrA _F | GCGCCACTTTCGCTTCTG |
| GyrA _R | GGTCTCCTTCGGCATCAACA |
| AcrA_F | CGTCATTGGTGACCAGCTCT |
| AcrA_R | TACACCAAAGTCACCTCGCC |
| AcrB_F | ACCGGTGATCGCTTCAACTT |
| AcrB_R | GAGGACCAGGGGGTATTCCT |
| TolC_F | TGCTCCCCATTCTTATCGGC |
| TolC_R | TTTTTCCTGCAGTGTCAGGGC |
| RamA_F | AAGACGCGGGTAAAGGTCTG |
| RamA_R | CCGCTCAGGTGATTGACACT |
| RamR_F | TGCGTCTGGTACGGATGATGA |
| RamR_R | CAGCTGGCACATTTCGTTGAG |
| RomA_F | TAGGTACCAGGAGGAGTGGC |
| RomA_R | GACGGCCAGTTCCATAACCA |
