# Supplementary material for: Phenotypic and genomic changes in enteric Klebsiella populations during long-term ICU patient hospitalization: the role of RamR regulation
Source: mSphere. 2024 Nov 29;9(12):e00704-24. doi: 10.1128/msphere.00704-24 (PMC11656808; doi:10.1128/msphere.00704-24)
Supplement: Table S3 — Demographics of the five patients with persistent KpSC carriage in the ICU long-term stay. [file msphere.00704-24-s0005.pdf]

Table S3: Demographics of the five patients with persistent KpSC carriage in the ICU long-term stay.
KpSC: *K. pneumoniae* species complex; ST: Sequence Type; ICU: Intensive Care Unit

	**BOMA**	**ESSO**	**ETJP**	**GUMA**	**OZAR**
**Age (years)**	31	61	66	76	41
**Sex**	Female	Female	Male	Female	Male
**Comorbidity**	None	None	None	None	None
**Reason of admission**	Cerebral bleeding	Cerebral bleeding	Digestive tract bleeding	Cerebral bleeding	Head trauma
**Days of ICU**	80	50	36	18	77
**Positive swab to KpSC (total swabs)**	7 (8)	7 (8)	4 (5)	2 (3)	3 (8)
**Number of strains isolated**	35	35	20	10	15
**Species**	*K. pneumoniae*	*K. variicola*	*K. pneumoniae*	*K. pneumoniae*	*K. variicola*
**ST (MLST)**	ST36	ST1563	ST405	ST163	ST1791
**Antibiotics in ICU**	Yes	Yes	Yes	Yes	Yes
**Outcomes**	Rehabilitation establishment	Neurosurgery	Other establishment	Neurology	Rehabilitation establishment
