# Supplementary material for: Phenotypic and genomic changes in enteric Klebsiella populations during long-term ICU patient hospitalization: the role of RamR regulation
Source: mSphere. 2024 Nov 29;9(12):e00704-24. doi: 10.1128/msphere.00704-24 (PMC11656808; doi:10.1128/msphere.00704-24)
Supplement: Table S10 — ESSO microdilution MICs to antibiotics and antiseptics. [file msphere.00704-24-s0007.pdf]

	Antibiotics MIC (mg/l)											Antiseptics MIC (mg/l)		
**Strains**	**TEM**	**TZP**	**CAZ**	**CRO**	**FOX**	**ERY**	**CHL**	**CST**	**NAL**	**CIP**	**TGC**	**Chlorhexidine**	**DDAC**	**Povidone iodine**
**ESSO2**	4	**64**	0.5	0.06	4	256	8	0.125	4	0.03	0.5	32	2	1563
**ESSO3**	8	**128**	0.5	0.06	4	256	8	0.250	4	0.03	0.5	32	2	1563
**ESSO4**	4	**128**	0.5	0.06	2	256	8	0.250	4	0.03	0.5	32	4	1563
**ESSO5**	4	**64**	0.5	0.06	4	256	8	0.125	4	0.03	0.5	16	2	3125
**ESSO6**	4	**128**	0.5	0.06	4	256	8	0.125	4	0.03	0.5	16	2	1563
**ESSO7**	**16**	**256**	2	0.125	**32**	512	**64**	0.125	**32**	0.125	4	32	2	1563
**ESSO8**	**16**	**256**	2	0.125	16	512	**64**	0.125	16	0.125	2	64	4	1563

Table S10: ESSO persistent KpSC microdilution MIC (mg/l). Lines in grey highlight strains with modification of AMR profile. Resistant MIC as defined by CA-SFM/EUCAST are highlighted in bold.

CAZ: ceftazidime , CHL: chloramphenicol, CIP: ciprofloxacin, CRO: ceftriaxone, CST : colistin, ERY: erythromycin, FOX: cefoxitin, NAL: nalidixic acid, TZP: piperacillin/tazobactam, TEM: temocillin, TGC: tigecycline, DDAC: didecyldimethylammonium chloride
